# Effect of Vietnamese Fly Ash on Selected Physical Properties, Durability and Probability of Corrosion of Steel in Concrete

**DOI:** 10.3390/ma12040593

**Published:** 2019-02-16

**Authors:** Chinh Van Nguyen, Paul Lambert, Quang Hung Tran

**Affiliations:** 1 Department of Civil Engineering, The University of Danang-University of Science and Technology, 54 Nguyen Luong Bang, Danang, Vietnam; nvchinh@dut.udn.vn (C.V.N.); tqhung@dut.udn.vn (Q.H.T.); 2 Materials and Corrosion Technology, Mott MacDonald, Altrincham WA14 1ES, UK

**Keywords:** concrete, fly ash, workability, compressive strength, acid resistance, probability of corrosion

## Abstract

Vietnamese fly ash was used as a partial replacement for ordinary Portland cement in the proportions of 10%, 20% and 40%, while the water to cementitious ratios were kept constant at 0.42, 0.5 and 0.55, respectively, for three groups. The compressive strengths of all mixes were determined up to 90 days. The acid resistance of fly ash concrete was examined by the mass loss and compressive strength loss of 100 × 100 × 100 mm^3^ cubes immersed in a 10% H_2_SO_4_ solution. The probability of steel corrosion in the fly ash concrete was assessed by measuring the half-cell potentials of steel bars within beams dimensions of 100 × 100 × 500 mm^3^, and the flexural strengths of these beams after 300 days of immersion in a 5% NaCl solution were determined. The results demonstrate that the compressive strength of fly ash concrete is reduced at an early age but increases as the concrete continues to hydrate. The fly ash increases the sulfuric acid resistance of concrete. Fly ash additions have only a limited effect on reducing the risk of probability of corrosion of steel in the concrete. The load capacities of 10% and 20% fly ash reinforced concrete beams are higher than that of the control beams after 300 days immersed in a 5% NaCl solution.

## 1. Introduction

The widespread use of concrete as a construction material has many associated environmental impacts related to its raw materials and manufacturing, most notably the production of Portland cement. By comparison, fly ash is a by-product of coal-fired electricity generation, but has the potential to be used as a supplementary cementitious material in concrete, replacing a proportion of the Portland cement [1,2].

Fly ash (FA) is classified by several different standards. Based on the source of origin and composition, ASTM C618, standard specification for coal fly ash and raw or calcined natural pozzolan for use in concrete [3] divides fly ash into two classes, namely Class F and Class C. The key difference between two classes is the amount of calcium, silica, alumina and iron content in the ash. Class F fly ash has a total calcium oxide content of typically 1%–12%, mostly in the form of calcium hydroxide, calcium sulfate, and a glassy component, in combination with silica and alumina. Class C fly ash has a high calcium oxide content of 30%–40%. An additional difference between fly ash Class F and Class C is that the amount of alkalis (combined sodium and potassium), and sulfate (SO_4_^2−^) are generally higher in the Class C fly ash than in the Class F fly ash [4]. Based on the calcium content of the fly ash, CAN/CSA A3001-03, cementitious materials for use in concrete [5] divides fly ash into three types. The calcium content of fly ash may be the best indicator of how effective the fly ash will be as a cement replacement in concrete [6], although other compounds including alkalis (Na_2_O and K_2_O), carbon (usually measured as loss on ignition) and sulfate (SO_3_) can also affect the performance of the fly ash.

The use of fly ash as a supplementary cementitious material (SCM) in concrete potentially has economic and performance advantages, including improved workability, enhanced mix efficiency and increased durability. Fly ash as a cement replacement is also widely recognised and specified in standards covering SCMs [3] and general purpose and blended cements [5]. More recently, the focus for the use of fly ash in concrete has shifted to quantifying the benefits offered in enhancing concrete sustainability [6]. Fly ash has been successfully used in Portland cement concrete and as a component of blended cements for more than 50 years. There are some notable structures in which fly ash has been used including the Prudential Building, the first skyscraper built in Chicago after World War II. In 1955, Lednock Dam in the UK used approximately 60,000 m^3^ of fly ash as a partial cement replacement of concrete with an estimated saving of 3,000 tonnes of ordinary Portland cement (OPC) [7].

Fly ash can directly contribute to sustainable development by reducing the quantity of Portland cement required while maintaining other criteria including engineering design aspects, constructional aspects and economic advantages [8]. This is due to the pozzolanic reaction of fly ash in concrete. Portland cement is a product of four mineralogical phases, which are tricalcium silicate, C_3_S (3CaO SiO_2_), dicalcium silicate, C_2_S (2CaO SiO_2_), tricalcium aluminate, C_3_A (3CaO Al_2_O_3_) and tetra-calcium alumino-ferrite, C4AF (4CaO Al_2_O_3_ Fe_2_O_3_). These compounds react with water to produce a hydrated calcium silicate (CSH) and lime. However, if fly ash is added to the mix, it reacts with the lime to form additional CSH, contributing to the cementing product produced by the normal hydration of the cement paste [9].

There has been significant research concerning the influence of different fly ashes on the physical properties and durability of concrete. The incorporation of fly ash into the mixture greatly influences the properties of the fresh concrete plus the mechanical properties and durability of the hardened concrete. The extent to which fly ash affects these properties is dependent not only on the level and composition of the fly ash, but also on other parameters including the composition and proportions of the other ingredients in the concrete mix, the type and size of the concrete component, the exposure conditions during and after placement and the optimum construction practices. Clearly, there is no single replacement level that is best suited for all applications [10]. The strength of fly ash concrete is affected by several factors including type, chemical composition, fineness and ratios of the fly ash and Portland cement. For example, Class C (high calcium) fly ash produces higher early strength than Class F (low calcium) fly ash, because it can initiate the necessary chemical reactions by its own lime content [11]. Moreover, high fineness of fly ash increases the density and pozzolanic activity of the concrete, resulting in an increase in concrete strength [11]. However, it has been concluded that no clear relationship between strength development and calcium content has been identified [12]. It also has been reported that the drying shrinkage of high-volume fly ash concrete is generally less than conventional concrete [13,14]. This is due to the reduced amounts of water required to produce such concrete [10].

This paper investigates the development of compressive strength, acid resistance via visual monitoring of the concrete surfaces, mass loss and compressive strength loss; and the reduced probability of corrosion for embedded steel for concrete made with OPC partially replaced by fly ash from Northern Vietnam in varying proportions of 0%, 10%, 20% and 40%, and varying water to cementitious material (W–CM) ratios of 0.42, 0.50 and 0.55.

## 2. Materials and Methods

### 2.1. Materials

The cementitious materials used in this study consisted of ordinary Portland cement from the Song Gianh Company, Quang Binh, Vietnam and ASTM Class F fly ash [3] from Pha Lai Power Station, Hai Duong, Vietnam. The physical properties and chemical compositions of the fly ash are given in Table 1. The fine aggregate was natural sand from the Ky Lam River, Quang Nam, Vietnam. The coarse aggregate was crushed limestone with a maximum nominal size of 20 mm from Phuoc Ly, Danang, Vietnam. 

### 2.2. Mixture Proportions and Specimens

The compositions of all mixes are presented in Table 2. The specimens were divided into three groups. Group 1 includes 4 mixes with a W–CM ratio of 0.42. The OPC was replaced by 10%, 20% and 40% fly ash (by weight) for M2, M3 and M4, respectively, while mix M1 was the control mix without any fly ash substitution. Group 2 consists of 4 mixes with a W–CM ratio of 0.5. The OPC was replaced by 10%, 20% and 40% fly ash (by weight) for M6, M7 and M8, respectively, while M5 was the control mix without any fly ash substitution. Group 3 consists of 4 mixes with a W–CM ratio of 0.55. The OPC was replaced by 10%, 20% and 40% fly ash (by weight) for M10, M11 and M12 respectively, while M9 was the control mix without any fly ash substitution. Details of the specimens and curing regime are given in the following sections.

### 2.3. Compressive Strength Test

The compressive strength tests were conducted in accordance with BS EN 12390-3:2009: testing hardened concrete—part 3: compressive strength of test specimens [15]. Eighteen cubes with dimensions of 100 × 100 × 100 mm^3^ were cast for each mix to determine the compressive strengths at 1, 7, 14, 28, 56 and 90 days. All specimens were demolded after one day and cured in water at approximately 27 °C until the test dates.

### 2.4. Acid Resistance Test

The acid resistance of the concrete mixes was evaluated in accordance with the modified ASTM C267 standard test methods for chemical resistance of mortars, grouts, and monolithic surfacings and polymer concretes [16]. Six cubes with dimensions of 100 × 100 × 100 mm^3^ were cast from which three cubes were used to determine the mass loss and residual compressive strength after immersion in a 10% sulfuric acid (H_2_SO_4_) solution for 90 days in the laboratory. The 10% sulfuric acid solution was made by dilution directly from 98% concentrated sulfuric acid with potable water. It is noted that 10% sulfuric acid does not represent a specific exposure environment; however, this concentration of acid has been used to test the sulfuric acid resistance of construction products by the Los Angeles County for over 20 years [17]. The relatively high concentration of 10% sulfuric acid was used to accelerate the experiments and provide results within an acceptable timescale. The 10% sulfuric acid solution was changed monthly. After 90 days of immersion in the solution, the cubes were taken out and weighed to determine loss of mass and tested for residual compressive strength. The three remaining cubes from each mix were used to determine the compressive strengths of the corresponding reference specimens cured in water.

### 2.5. Probability of Corrosion of Steel in Fly Ash Concrete and Load Capacities of Fly Ash Reinforced Concrete Beams

**Figure 1:**
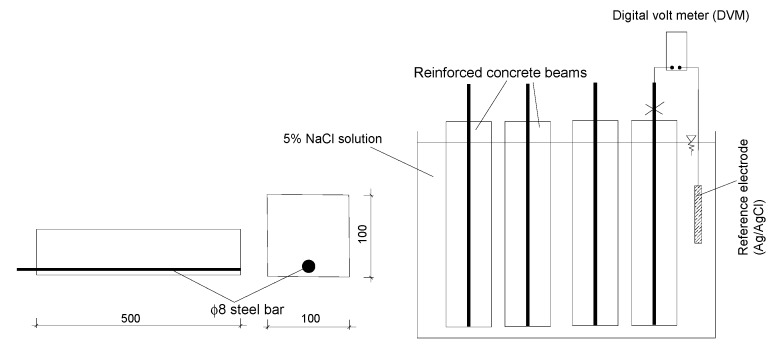
Beam details and schematic half-cell potential measurement of embedded steel bars.

The probability of steel corrosion in concrete with and without fly ash was investigated on twelve beams with a cross section of 100 × 100 mm^2^ and a length of 500 mm with one 8 mm diameter steel bar of grade 250, with a yield strength of 250 MPa, located towards the lower face of the beams with a reinforcement cover of 15 mm (see Figure 1). After 28 days of curing in water, the beams were transferred to a 5% NaCl solution simulating a marine environment where the exposed air/saline water interface creates an environment to corrosion [18]. The 5% NaCl solution was changed monthly.

The half-cell potentials of the steel bars were monitored and recorded until the termination of the testing at 301 days. Half-cell potential was used to quantify the progression of corrosion activity in general accordance with ASTM C876 [19]. This method measures the difference of electrical potential between the steel bars in the test specimens and a standard reference electrode. The reference electrode used in this test was Ag/AgCl/0.5 M KCl. In accordance with ASTM C876, when the half-cell potential (vs. Ag/AgCl/0.5 M KCl) is in the range of −100 to −250 mV, the probability of corrosion is assessed as uncertain; when the half-cell potential is less negative than −100 mV (vs. Ag/AgCl/0.5 M KCl), the probability of corrosion activity is considered to be low (less than 10%); and when the half-cell potential is more negative than −250 mV, the probability of corrosion activity may be assumed to high (greater than 90%). These figures assume corrosion is due to chloride ions and that the cathodic reaction is not suppressed due to reduced oxygen, that is, as a result of the specimens being water saturated.

**Figure 2:**
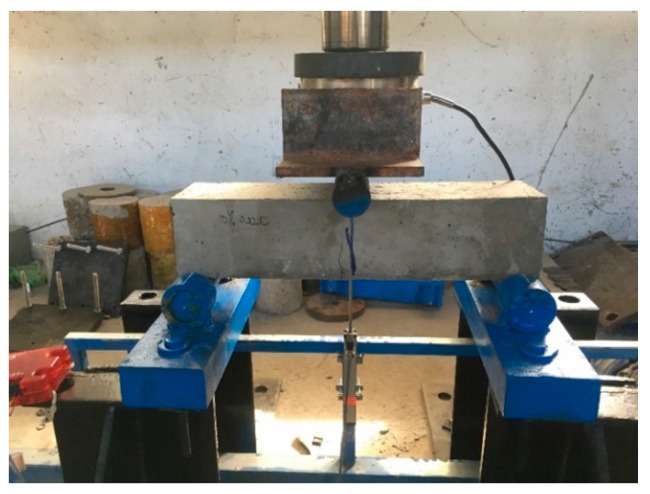
Three point bending test.

Half-cell potential is a simple but limited method of corrosion assessment that only provides information on the probability of corrosion activity but does not give any indication of the rate of metal loss, which can be achieved by using more advanced techniques, such as linear polarization to provide a comprehensive assessment of the corrosion activity of steel in concrete. The half-cell potential is however useful for identifying the initiation of corrosion activity as the basis for further investigation. After 301 days of immersion in 5% NaCl solution, all the reinforced concrete beams were tested under flexure to determine the ultimate load by a three point bending test (see Figure 2).

## 3. Results and Discussion

### 3.1. Workability of Fresh Concrete

**Figure 3:**
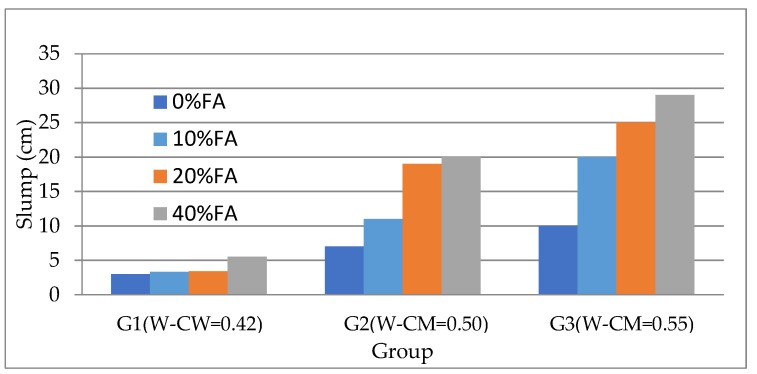
Slump of all fresh concrete mixes.

Slump tests were conducted immediately after mixing, and the results are given in Table 2 and Figure 3. It is apparent that for each group of specimens, fly ash substitution contributed to an increase in workability as evidence by the increasing slump. For Group 1 (W–CM = 0.42), the slump increased from 3 to 5.5 cm as the OPC was substituted by increasing percentages of fly ash up to 40%. For the same incremental increase in fly ash substitution, the slump for Group 2 (W–CM = 0.5) increased from 7 to 20 cm and for Group 3 (W–CM = 0.55) increased from 10cm to 29cm. This has previous been explained by the differences between the spherical shape of the fly ash particles and angular shape of the cement particles [20]. This again confirmed that the fly ash contributed to the increase of workability of the concrete. 

### 3.2. Compressive Strength Development

**Figure 4:**
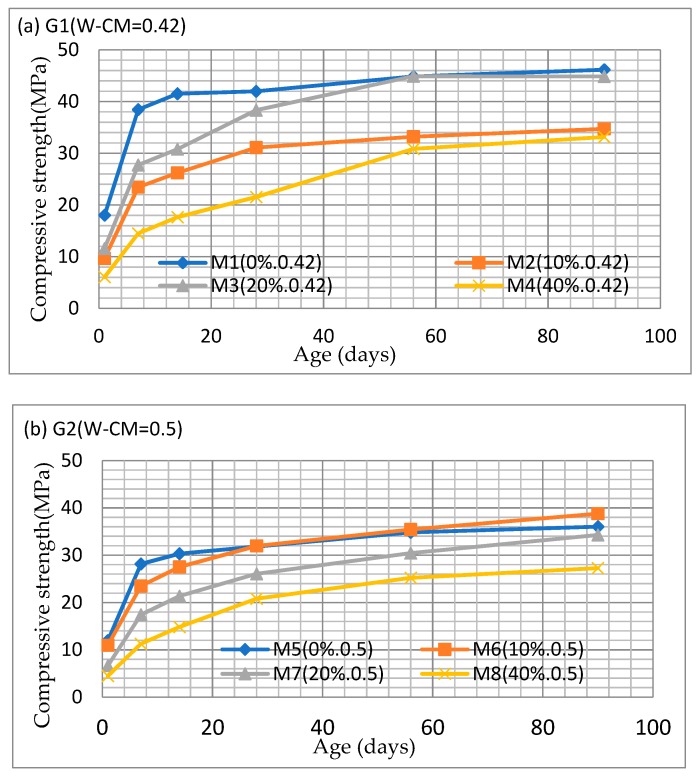

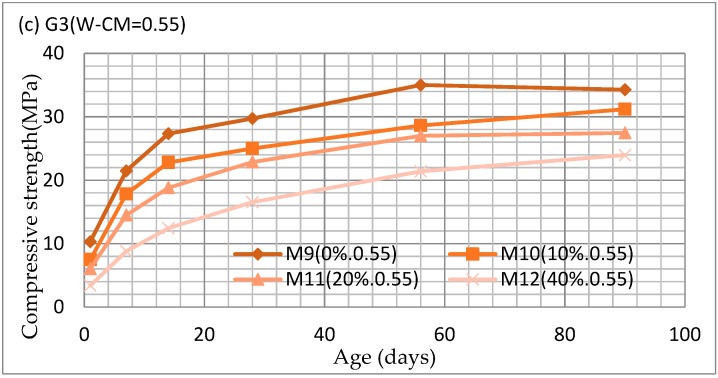
Compressive strengths of concrete specimens (**a**) G1 (W–CM = 0.42); (**b**) G2 (W–CM = 0.50); (**c**) G3 (W–CM = 0.55).

The compressive strengths of the various mixes are shown in Figure 4a–c. It can be seen that the compressive strengths of all the fly ash concrete specimens continued to develop until 90 days when they were tested and it can be inferred from the results that the strengths would have continued to increase further, although the rate of development is slower than at early age. By comparison, the compressive strengths of the control specimens (0% fly ash) for all W–CM ratios would not appear to have increased significantly after 90 days. 

Compared to the controls, the specimens with partial fly ash replacement show a reduction in the development of compressive strength at early age; however the long-term strength continues to develop and the compressive strength of some of the fly ash concrete specimens exceeded that of the control specimens, depending on the W–CM. For example, the compressive strength of concrete specimens M3 (20% fly ash, W–CM = 0.42) was almost equal to the compressive strength of control specimens M1 (0% fly ash, W–CM = 0.42) at 56 days (Figure 4a). The compressive strength of specimens with a 10% fly ash replacement and W–CM of 0.5 was higher than that of the control specimens at 28 days (Figure 4b). For Group 3 (W–CM = 0.55), although after 90 days, the compressive strengths of the fly ash specimens were lower than that of the control specimens, and the trend in strength gain suggests that the compressive strength of fly ash specimens can continue developing beyond that of the control specimens (Figure 4c). 

### 3.3. Acid Resistance of Fly Ash Concrete

#### 3.3.1. Visual Inspection

**Figure 5:**
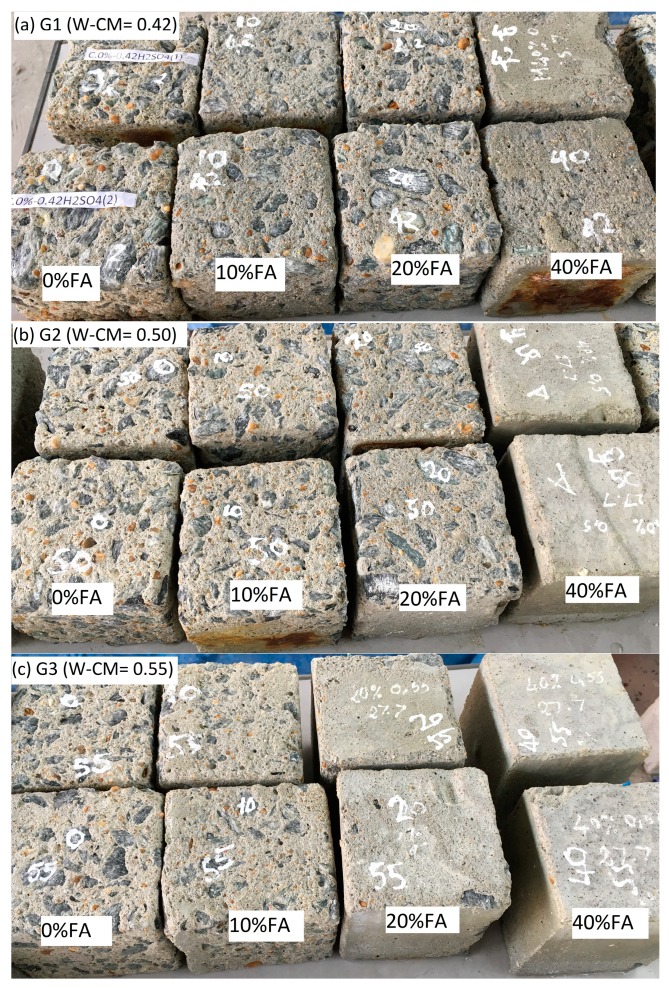
Surface appearance of concrete cubes after immersion in 10% sulfuric acid solution. (**a**) G1 (W–CM = 0.42); (**b**) G2 (W–CM = 0.50); (**c**) G3 (W–CM = 0.55).

After 90 days of immersion in a 10% sulfuric acid solution, the surfaces of all concrete cubes were cleaned and appear as shown in Figure 5. It can be seen that the fly ash concretes showed improved resistance to the degradation of concrete due to an attack by the sulfuric acid solution. The higher the percentage of fly ash replacement, the greater the apparent resistance to concrete surface degradation. A visual comparison can be made of the surfaces of the specimens in all three specimens groups (Figure 5a–c) as the level of fly increases from 0% to 40%. 

Counter-intuitively, as the water to cementitious ratio increased the resistance to 10%, the sulfuric acid solution by the fly ash containing specimens also increased. For example, comparison of the M4 (40% fly ash, W–CM = 0.42) and M8 (40% fly ash, W–CM = 0.5) specimens (see Figure 5) show the surface of the M4 specimen had visually degraded while the surface of the M8 specimen remained visually unchanged. Similarly, comparisons can be made for the M7 (20% fly ash, W–CM = 0.5) and M11 (20 fly ash, W–CM = 0.55) specimens. The influence of the water to cementitious ratios on the degradation of surface concrete is confirmed by the mass loss determinations, discussed in the following section.

#### 3.3.2. Mass Loss and Compressive Strength Loss

**Figure 6:**
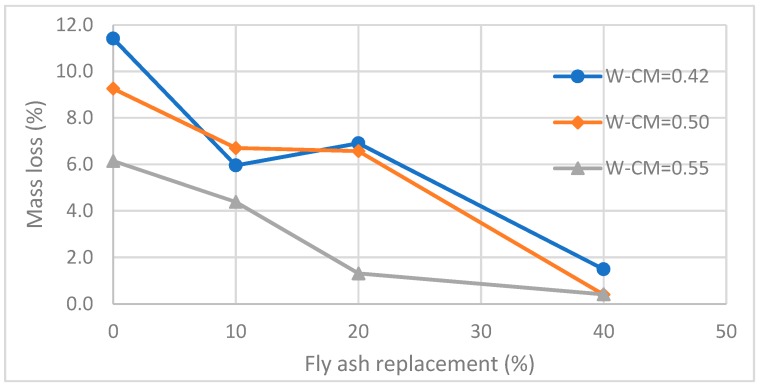
Relationships between mass loss and proportion of fly ash replacement

**Figure 7:**
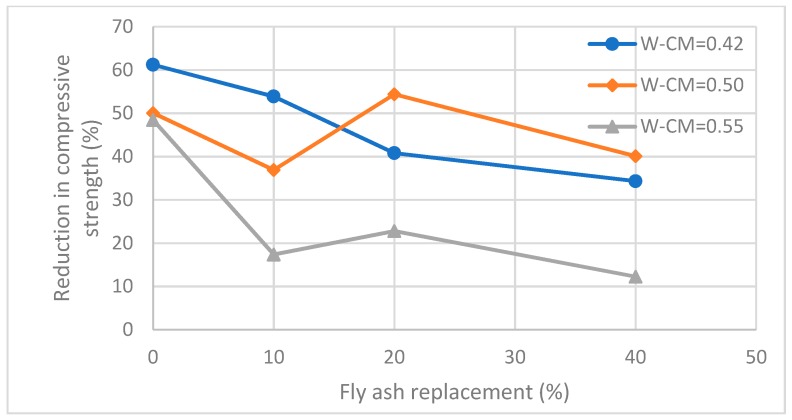
Relationships between compressive strength loss and proportion of fly ash replacement.

The change in mass and compressive strength for specimens exposed to 10% sulfuric acid solution are shown in Table 3 and Figure 6 and Figure 7. Figure 6 shows the mass losses of the control and fly ash substituted concrete cubes before and after immersion in the sulfuric acid solution. It is apparent that fly ash substitution reduced the mass loss, confirming an increase in the acid resistance. The higher fly ash replacement proportion, the greater the apparent acid resistance. For example, for specimens with a W–CM = 0.42, the mass losses of 0%, 10%, 20% and 40% fly ash replacement specimens are 11.4%, 6.0%, 6.9% and 1.5%, respectively. When W–CM = 0.5, the mass losses of 0%, 10%, 20% and 40% fly ash replacement specimens are 9.3%, 6.7%, 6.6% and 0.4%, respectively. When W–CM = 0.55, the mass losses of 0%, 10%, 20% and 40% fly ash replacement specimens are 6.1%, 4.4%, 1.3% and 0.4%, respectively.

Similar to surface degradation of the concrete, the mass loss reduced when W–CM ratios increased. For example, at a 0% fly ash replacement, the mass losses of the concrete specimens are 11.4%, 9.3% and 6.1% for W–CM ratios of 0.42, 0.5 and 0.55, respectively. At a 10% fly ash replacement, the mass losses of the concrete specimens are 6%, 6.7% and 4.4% for W–CM ratios of 0.42, 0.5 and 0.55, respectively. At a 20% fly ash replacement, the mass losses of the concrete specimens are 6.9%, 6.6% and 1.3 % for W–CM ratios of 0.42, 0.5 and 0.55, respectively. At a 40% fly ash replacement, the mass losses of the concrete specimens are 1.5%, 0.4% and 0.4 % for W–CM ratios of 0.42, 0.5 and 0.55, respectively. It can be concluded that fly ash reduces the mass loss from the concrete surface resulting from sulfuric acid exposure, and the higher the proportion of fly ash replacements, the lower the mass loss from the concrete surface. Moreover, the mass loss also depends on the water to cementitious ratio, with higher W–CM ratios resulting in lower mass loss.

Figure 7 shows the relationships between the compressive strengths loss due to acid exposure and fly ash replacement proportions. It is apparent that the partial replacement with fly ash reduced the compressive strength losses of the concrete, with greater fly ash replacement proportions resulting in lower compressive strength loss, indicating that fly ash additions increase the acid resistance of concrete. For W–CM = 0.42, the compressive strength losses are 61.2%, 53.9%, 40.8% and 34.3% for 0%, 10%, 20% and 40% fly ash replacement specimens, respectively. For W–CM = 0.5, the compressive strength losses are 50.1%, 36.9%, 54.4% and 40.1% for 0%, 10%, 20% and 40% fly ash replacement specimens, respectively. For W–CM = 0.55, the compressive strength losses are 48.5%, 17.4%, 22.8% and 12.2% for 0%, 10%, 20% and 40% fly ash replacement specimens, respectively.

As previously observed with respect to surface degradation and mass loss, at a 0% and 10% fly ash replacement, the compressive strength loss reduced when W–CM ratios increased. For example, the compressive strengths loss of concrete with a 0% fly ash replacement are 61.2%, 50.1% and 48.5% for W–CM ratios of 0.42, 0.5 and 0.55, respectively. The compressive strengths losses of concrete at a 10% fly ash replacement are 53.9%, 36.9% and 17.4% for W–CM ratios of 0.42, 0.5 and 0.55 respectively. At 20% and 40% fly ash replacement, the compressive strengths losses of concrete for W–CM = 0.5 are higher than that for W–CM of 0.42 and 0.55. In general, fly ash decreases the compressive strength loss resulting from sulfuric acid exposure demonstrating an increased acid resistance. The greater the level of fly ash replacement, the lower the loss in compressive strength. Within the scope of this investigation, a W–CM ratio of 0.55 demonstrates the highest acid resistance, compared to W–CMs of 0.42 and 0.5.

### 3.4. Probability of Corrosion of Steel in Fly Ash Concrete and Load Capacities of Fly Ash Reinforced Concrete Beams

#### 3.4.1. Half-Cell Potential

**Figure 8:**
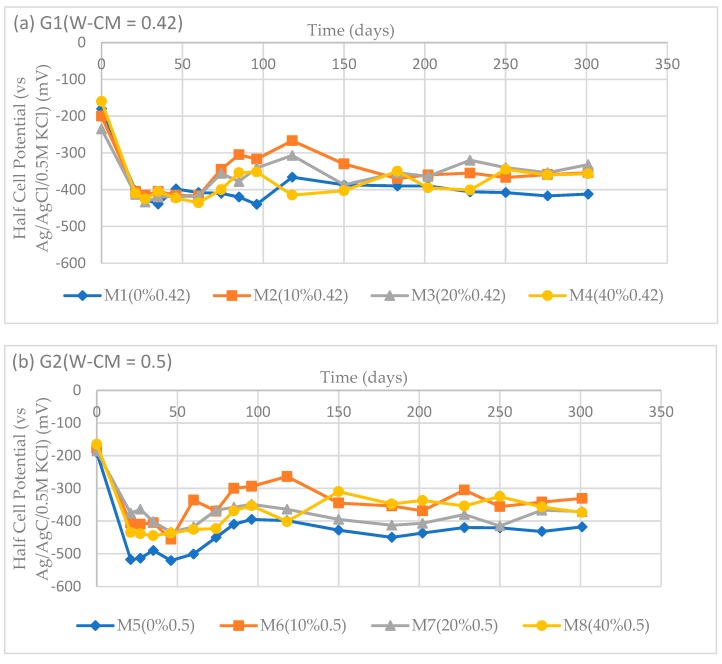

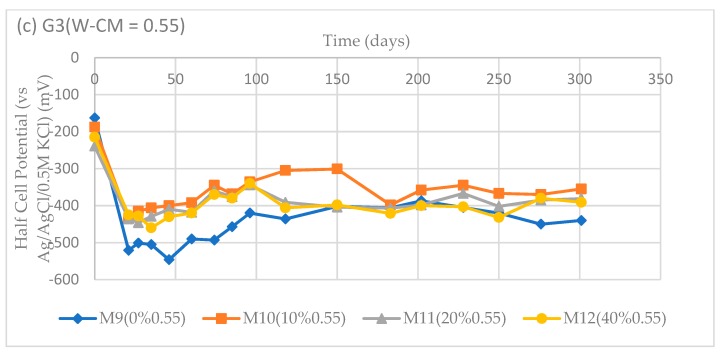
Half-cell potentials of steel bars. (**a**) G1 (W–CM = 0.42); (**b**) G2 (W–CM = 0.50); (**c**) G3 (W–CM = 0.55).

Half-cell potentials of the embedded steel bar specimens of Groups 1, 2 and 3 are plotted in Figure 8a–c, respectively. In general, the half-cell potentials of all steel bars changed from their passive state at around –150 mV (vs. Ag/AgCl/0.5M KCl) to more negative than –400 mV (vs. Ag/AgCl/0.5M KCl). For Group 1 (W–CM = 0.42), the half-cell potential of the steel bar made of a 10% fly ash replacement was less negative than that of 20%, 40% and 0% fly ash replacement, indicating a lower risk of corrosion. This means that for W–CM = 0.42, 10%FA showed more corrosion resistance than other samples. For Group 2 (W–CM = 0.5), the half-cell potential of the steel bar made of 10% fly ash concrete was also less negative than that of 20%, 40% and 0% fly ash concrete, indicating a better corrosion resistance. The results is repeated at W–CM = 0.55 (Group 3) as the half–cell potential of steel bar in 10% fly ash concrete was again less negative than that of 20%, 40% and 0% fly ash replacement. 

The half-cell potentials of all the steel bars were more negative than −250 mV after 21 days of immersion in a 5% NaCl solution, consistent with a high probability of corrosion activity. Within the scope of this investigation fly ash showed little beneficial effect on the initial corrosion resistance of steel bars in concrete, with the 10% fly ash replacement appearing to have the most positive effect.

#### 3.4.2. Flexural Resistance of Fly Ash Reinforced Concrete Beams

After 301 days of immersion in 5% NaCl solution, all beams were tested in flexure under three point bending. The yield and ultimate loads of all beams were determined, and the results are presented in Table 4. Table 4 shows that for Group 1, the yield load and ultimate load of the control beam (0% fly ash) were higher than that of the fly ash replacement beams. The yield load of the control beam was 21 kN while the yield load of 10%, 20% and 40% fly ash replacement beams were 15.5, 16.6 and 16.3 kN, respectively. The ultimate failure load of the control beam was 21.55 kN, while the ultimate failure loads of 10%, 20% and 40% fly ash replacement beams were 16.23, 18.9 and 17.08 kN, respectively.

Although earlier results indicate that the compressive strengths of concrete made with partial fly ash replacement can match that of control concrete without fly ash after 90 days (Section 3.2), the load capacity of the control beam without a fly ash replacement remained higher than that of the fly ash replacement beams after more than 300 days immersed in 5% NaCl. A possible explanation for this observation is that more extensive corrosion occurring in the control beams may increase the bond at steel and concrete interface.

For Group 2 (W–CM = 0.5), the yield load of the control beam remained higher than that of the fly ash beams. The yield load of the control beam was 16.00 kN, while the yield load of the 10%, 20% and 40% fly ash replacement beams were 17.00, 16.2 and 15.8 kN, respectively. However, the ultimate load of the 10% and 20% fly ash replacement beams were higher than that of the control beam while the ultimate load of the 40% fly ash beam was smaller than that of the control beam (0% fly ash). The ultimate failure load of the control beam was 17.32 kN, while the ultimate failure loads for 10%, 20% and 40% fly ash replacement beams were 18.45, 17.97 and 16.95 kN, respectively. This trend is consistent with that previously observed for the development of compressive strength of fly ash concrete in Section 3.2. This also demonstrates that the increase in the load capacity of 10% and 20% fly ash reinforced concrete beams depend on the compressive strength of the concrete rather than the corrosion of the steel, as the severity and extent of the corrosion of these beams when immersed in 5% NaCl for 301 days was not significant.

For Group 3 (W–CM = 0.55) the yield load and ultimate load of the fly ash replacement beams were higher than that of the control beam. The yield load of the control beam was 14 kN, while the yield load of the 10%, 20% and 40% fly ash replacement beams were 18, 14.5 and 15.5 kN, respectively. The ultimate failure load of the control beam was 16.71 kN, while the ultimate failure loads for the 10%, 20% and 40% fly ash replacement beams were 19.02, 17.66 and 18.73 kN, respectively. It can be seen that the load capacity of the Group 3 beams are consistent with the trend in compressive strength development (Figure 3).

## 4. Conclusions

Based on the results reported in this paper, the following conclusions can be made:

- The Vietnamese sourced fly ash investigated in this study contributes to improvements in the workability of fresh concrete. This is consistent with differences between the spherical shape of fly ash particles and angular shape of the cement particles, as confirmed by previous research.
- Within the range of investigation (10% to 40% replacement), fly ash reduced the compressive strength of the concrete at early age, either before 28 days or 56 days depending on the W–CM ratios. After this time, the compressive strength of the fly ash specimens continues to develop, albeit slowly, and gains higher strengths than that of the control specimens, while the compressive strengths of the control specimens (0% fly ash) is no longer developed.
- Within the range of investigation, (10% to 40% replacement), the optimum fly ash replacement proportion was found to be 20% by weight of Portland cement.
- Fly ash increases the sulfuric acid resistance of the concrete. This results in a reduction in surface degradation, mass loss and compressive strength loss resulting from exposure to sulfuric acid solution. The greater the level of fly ash replacement, the less surface degradation, mass loss and compressive strength loss was observed. Within the scope of this investigation, water to cementitious ratio of 0.55 shows the highest acid resistance compared with values of 0.42 and 0.5.
- Within the scope of this investigation, fly ash has had little effect on reducing the probability of corrosion of steel bars embedded in concrete specimens, but a 10% fly ash replacement showed greatest benefit.
- In general, 10% and 20% fly ash reinforced concrete beams show better flexural resistance than that of the control beams without fly ash after 300 days exposure in a 5% NaCl solution.
- Further research should be conducted at different levels of replacements (e.g., 5%, 15%, 25%, 30%, 35%) to identify optimum replacement levels for the enhancement of the various performance criteria investigated.

## Figures and Tables

**Table 1:** The physical properties and chemical compositions of fly ash.

Properties	Fly Ash
Fineness (%)	21.5 (>45 μm)
Loss on ignition (LOI) (%)	5.83
Moisture (%)	0.04
SiO_2_ (%)	58.9
Fe_2_O_3_ (%)	5.75
Al_2_O_3_ (%)	23.9
SO_3_ (%)	0.03

**Table 2:** Mix proportions for the concretes used in the preparation of the specimens.

Group	Identification	W–CM	W–C	OPC	Fly Ash	OPC + Fly Ash	Aggregate	Sand	Slump (cm)
G1	M1 (0% 0.42)	0.42	0.42	1	0	1	3	2	3
	M2 (10% 0.42)	0.42	0.47	0.9	0.1	1	3	2	3.3
	M3 (20% 0.42)	0.42	0.53	0.8	0.2	1	3	2	3.4
	M4 (40% 0.42)	0.42	0.7	0.6	0.4	1	3	2	5.5
G2	M5 (0% 0.50)	0.5	0.5	1	0	1	3	2	7
	M6 (10% 0.50)	0.5	0.56	0.9	0.1	1	3	2	11
	M7 (20% 0.50)	0.5	0.63	0.8	0.2	1	3	2	19
	M8 (40% 0.50)	0.5	0.83	0.6	0.4	1	3	2	20
G3	M9 (0% 0.55)	0.55	0.55	1	0	1	3	2	10
	M10 (10% 0.55)	0.55	0.61	0.9	0.1	1	3	2	20
	M11 (20% 0.55)	0.55	0.69	0.8	0.2	1	3	2	25
	M12 (40% 0.55)	0.55	0.92	0.6	0.4	1	3	2	29

**Table 3:** Mass loss and compressive strength loss of concrete.

Group	Specimen ID	Mass before Immersion in H_2_SO_4_ (g)	Mass after Immersion in H_2_SO_4_ (g)	Mass Loss (%)	Crushing Load Cured in Water (kN)	Crushing Load Cured in H_2_SO_4_ (kN)	Compressive Strength Loss (%)
1	M1 (0% 0.42)	2537.5	2248	−11.4	467.43	181.185	−61.2
	M2 (10% 0.42)	2469	2322	−6.0	364.815	168.195	−53.9
	M3 (20% 0.42)	2504	2331	−6.9	370.795	219.51	−40.8
	M4 (40% 0.42)	2524.5	2487	−1.5	336.465	220.97	−34.3
2	M5 (0% 0.50)	2522.5	2289	−9.3	410.255	204.885	−50.1
	M6 (10% 0.50)	2483	2316.5	−6.7	338.1	213.305	−36.9
	M7 (20% 0.50)	2548.5	2381	−6.6	334.235	152.385	−54.4
	M8 (40% 0.50)	2526.5	2516.5	−0.4	312.055	186.865	−40.1
3	M9 (0% 0.55)	2530	2374.5	−6.1	359.9	185.51	−48.5
	M10 (10% 0.55)	2531	2420	−4.4	242.795	200.64	−17.4
	M11 (20% 0.55)	2517	2484	−1.3	298.23	230.23	−22.8
	M12 (40% 0.55)	2445	2435	−0.4	247.515	217.2	−12.2

**Table 4:** Yield load and ultimate load of beams.

Group	Beam ID	Dimensions (mm^3^)	Yield Load (kN)	Ultimate Load (kN)
1	M1 (0% FA, 0.42)	100 × 100 × 500	21	21.55
	M2 (10% FA, 0.42)	100 × 100 × 500	15.5	16.23
	M3 (20% FA, 0.42)	100 × 100 × 500	16.6	18.9
	M4 (40% FA, 0.42)	100 × 100 × 500	16.3	17.08
2	M5 (0% FA, 0.50)	100 × 100 × 500	16	17.32
	M6 (10% FA, 0.50)	100 × 100 × 500	17	18.45
	M7 (20% FA, 0.50)	100 × 100 × 500	16.2	17.97
	M8 (40% FA, 0.50)	100 × 100 × 500	15.8	16.95
3	M9 (0% FA, 0.55)	100 × 100 × 500	14	16.71
	M10 (10% FA, 0.55)	100 × 100 × 500	18	19.02
	M11 (20% FA, 0.55)	100 × 100 × 500	14.5	17.66
	M12 (40% FA, 0.55)	100 × 100 × 500	15.5	18.73
